# Dr. Winn Read a Paper on the Elasticity of Arteries Considered as a Cause of Animal Heat

**Published:** 1854-07

**Authors:** 


### PHYSIOLOGICAL SOCIETY OF LONDON.

Dr. Winn read a paper on the *Elasticity of Arteries considered as a cause of Animal Heat.*

About seventeen years since, whilst making some experiments with caoutchouc, he (Dr. Winn) was forcibly struck with the property it possesses of evolving heat when suddenly elongated, and was led at the time to infer the probability of other bodies being similarly endowed. The elastic coat of arteries, especially, appeared to be one of the substances likely to exhibit this calefactory principle, and in the event of this being the case, he thought it would not be unreasonable to conclude that the incessant contradictions and dilatations of the arteries during life must form an efficient source of animal heat. Three years subsequently he was induced to resume the subject, and upon making an experiment with part of the aorta of a bullock, he was much gratified in being able to verify his previous conjecture. The experiment was performed as follows: Having cut off a circular portion of the descending arch of the aorta, above an inch in length, he laid it open, and carefully removed its external and internal coverings. He then pulled it to and fro with a continuous jerking motion (in imitation of the systole and diastole of the heart) for the space of about a minute. Immediately on discontinuing this movement, he placed it in the bulb of a thermometer, when he had the satisfaction of noticing, after a period of about two minutes, that the mercury had risen as many degrees. On removing the thermometer the heat diminished rapidly. To be certain that the increment of heat was not derived from any other source than that in question, he took the precaution of covering his finger with a double layer of flannel, to prevent the communication of heat from the body; he also covered his mouth with a handkerchief, to guard against the warm breath affecting the thermometer, whilst watching the progress of the experiment. It was also right to mention that the experiment was performed in a room without a fire, the temperature of the air being 55°. There were several difficulties to contend with during the investigation. The chief impediment appeared to be the moisture of the artery, which, by its evaporation, had a tendency to carry off a portion of the heat. However, by carefully drying the artery with a cloth, he succeeded in obviating this difficulty to a considerable extent, and was enabled to perform the experiment twice consecutively in a satisfactory manner. He had also, within the last fortnight, repeated the experiment in the presence of a medical friend with an equally satisfactory result. His attention was often arrested, whilst conducting the experiments, by other mechanical analogies between caoutchouc and the elastic coat of arteries. Like the former, the latter could be elongated to twice its ordinary length, and on suddenly stopping the tension, would return to its usual dimensions with considerable force and a snapping noise. From the preceding observations, Dr. Winn concluded that the generation of animal heat could now be fully and satisfactorily explained. Physiologists, after having clearly proved that a great portion of animal heat was the result of chemical changes in the blood, yet confessed that a residuum of heat could not be referred to this source; this residuum, he considered, arose from the mechanical action of the arteries. It would be exceedingly difficult to determine the precise quantity of heat given off during each beat of the artery; but if the development of only a very small quantity was admitted, it necessarily followed, from the circumstance of the action of the arteries being in incessant operation during life, that the heat must quickly accumulate to a great extent; and it is even probable that the body, unless cooled by the functions of the skin and lungs, would, in a short space of time, become preternaturally hot. The following physiological and pathological facts appeared to corroborate the views he had taken as to the mechanical source of heat: 1st. The minute distribution of the arteries to every part of the system ensured a general and equal distribution of heat. 2d. The *rigidity* of the arteries in old age was a probable cause of the diminution of animal heat at the close of life. 3d. The increased warmth of the body after exercise seemed to be readily explicable upon the principle of increased force of the arteries. 4th. In many diseases of the lungs, when their functions were at fault, and at a time when the arteries were beating with great strength and velocity, the heat of the body was found to be above the usual standard. 5th. Medicines which diminished the action of the heart and arteries almost invariably reduced the temperature of the body. 6th. The heat of local inflammation, in cases where the constitution did not sympathize to any extent, cannot be satisfactorily referred to any other source, as the arteries immediately in the neighborhood of the affected part are often throbbing violently, when the capillaries, which are supposed to play so important a part in the chemical theory, are generally considered to have their action impeded. His (Dr. Winn's) friend, Dr. Crisp, has hinted that many cold-blooded animals are remarkable for the great elasticity of their arteries. This fact could not affect his theory. The langor of the circulation in this class of animals more than counterbalances any calefactory effect which might otherwise be produced by the resiliency of their arterial structure. With respect to the nature of the mechanical force he had been investigating, little could be said. It might possibly be a little molecular friction; it was clearly, however, of a different nature from ordinary friction, which had also been considered a cause of animal heat, but Dr. Winn thought erroneously so, inasmuch as there is found every where, on examining the mechanism of the human frame, that the most efficient means of defence have been provided against its effects, as seen in the various synovial and serous membranes, &c.

Dr. O. Ward remarked, that the restoration of animal heat on recovery from asphyxia was more easily explicable by the chemical theory of animal heat than by Dr. Winn's theory.

Dr. E. Smith considered that Dr. Winn had brought forward a certain amount of "positive information," which was so far entitled to consideration. He instanced the heat derived from blushing as a phenomenon which could not be explained satisfactorily by the chemical theory.

Dr. Snow gave Dr. Winn credit for having developed an entirely "new idea." He, however, reminded Dr. Winn that the only force upon which the dilatation of the arteries depended was derived from the heart.

Dr. Pavey instanced the remarkable accession of heat and redness on one side of the head and neck, produced by division of the sympathetic nerve on one side of the body. This experiment militated against the correctness of the prevalent theory of animal heat.

Dr. O'Connor said that blushing had been attributed by Dr. Burgess to the influence of nervous action.

Dr. M'Donald suggested that Dr. Winn's experiment would be more satisfactory if the artery were elongated by metal appliances than by the fingers.

Dr. Winn, in his reply, observed that Dr. Ward had forgotten that simultaneous with the restoration of the respiration, in the cases to which he had referred, was the renewal of the heart's action. Dr. Winn felt indebted to Drs. Smith and Pavey for their interesting observations on blushing, and the effect of dividing the sympathetic nerves—observations which strongly corroborated the truth of his (Dr. Winn's) theory.—*London Lancet.*

